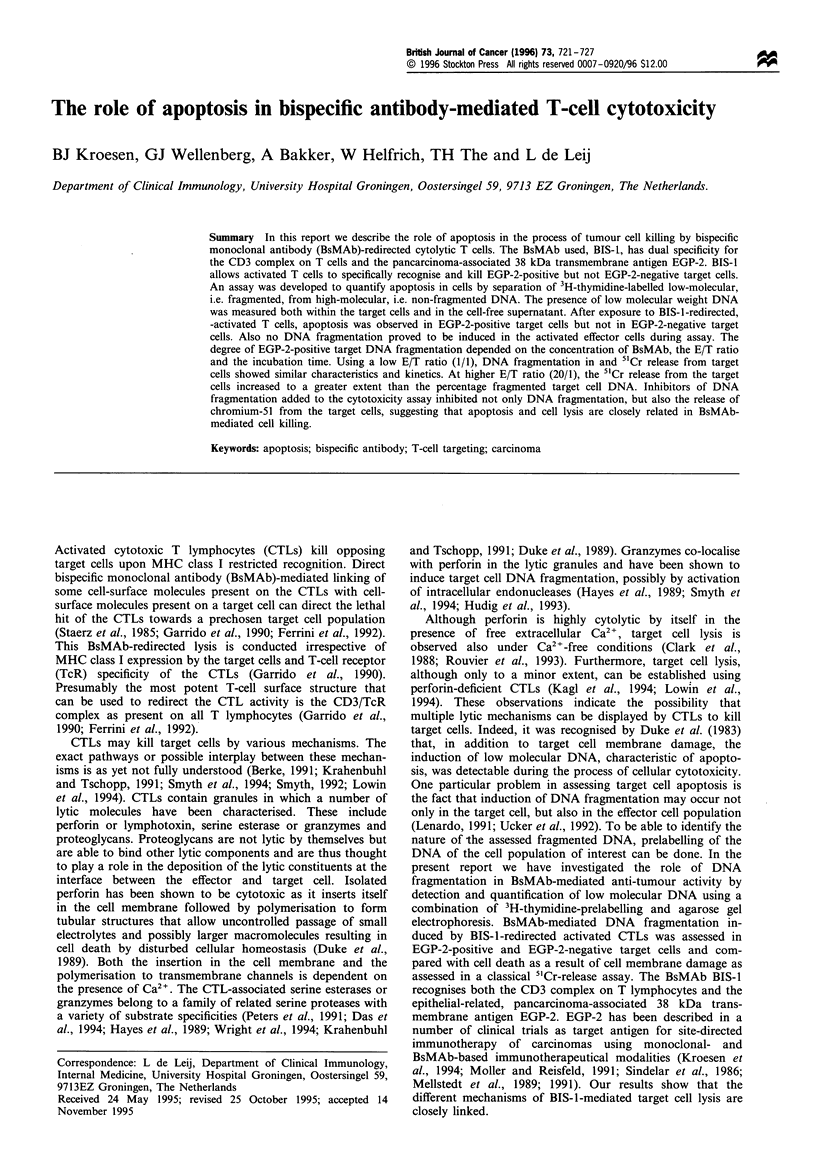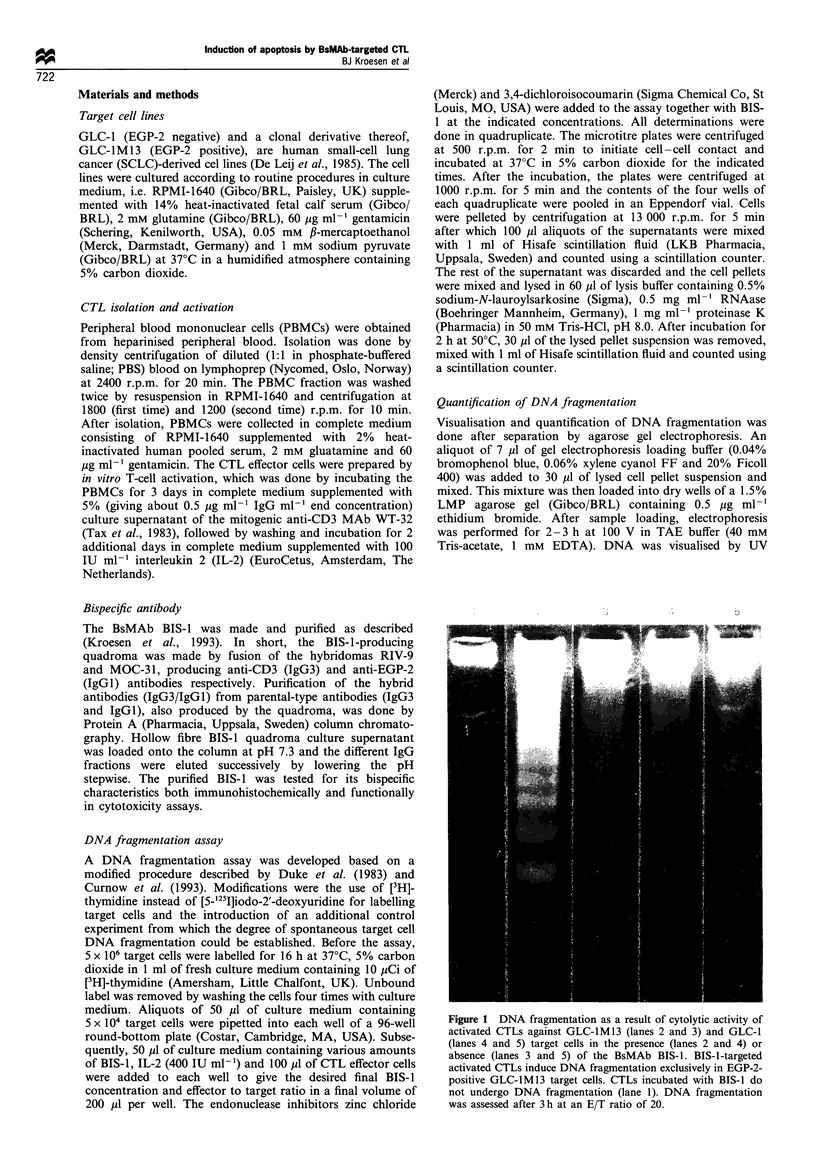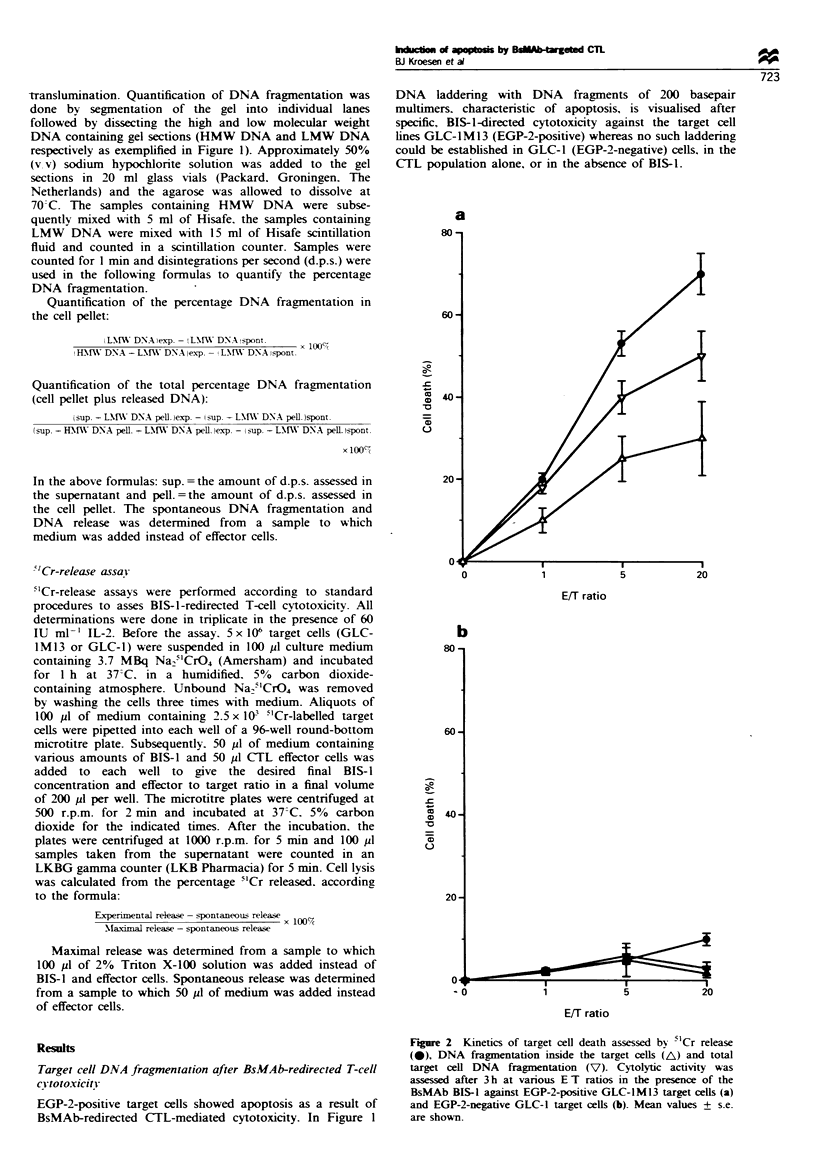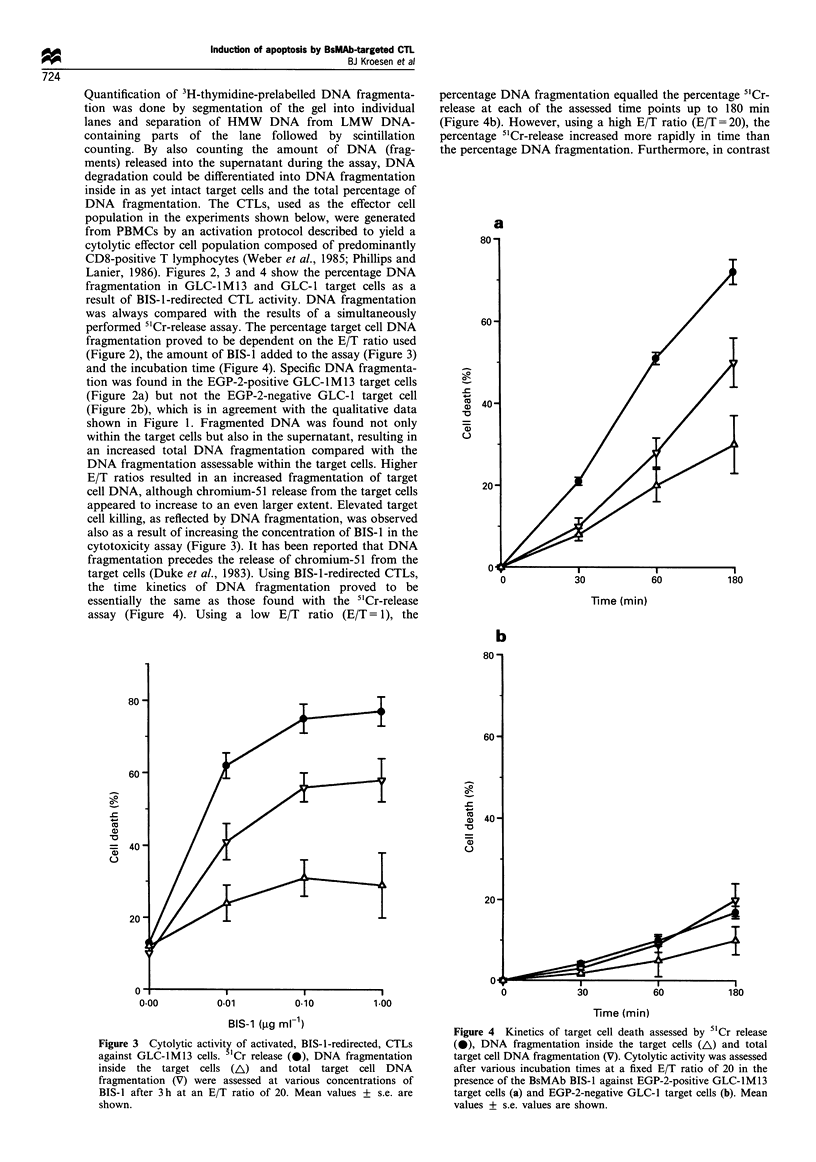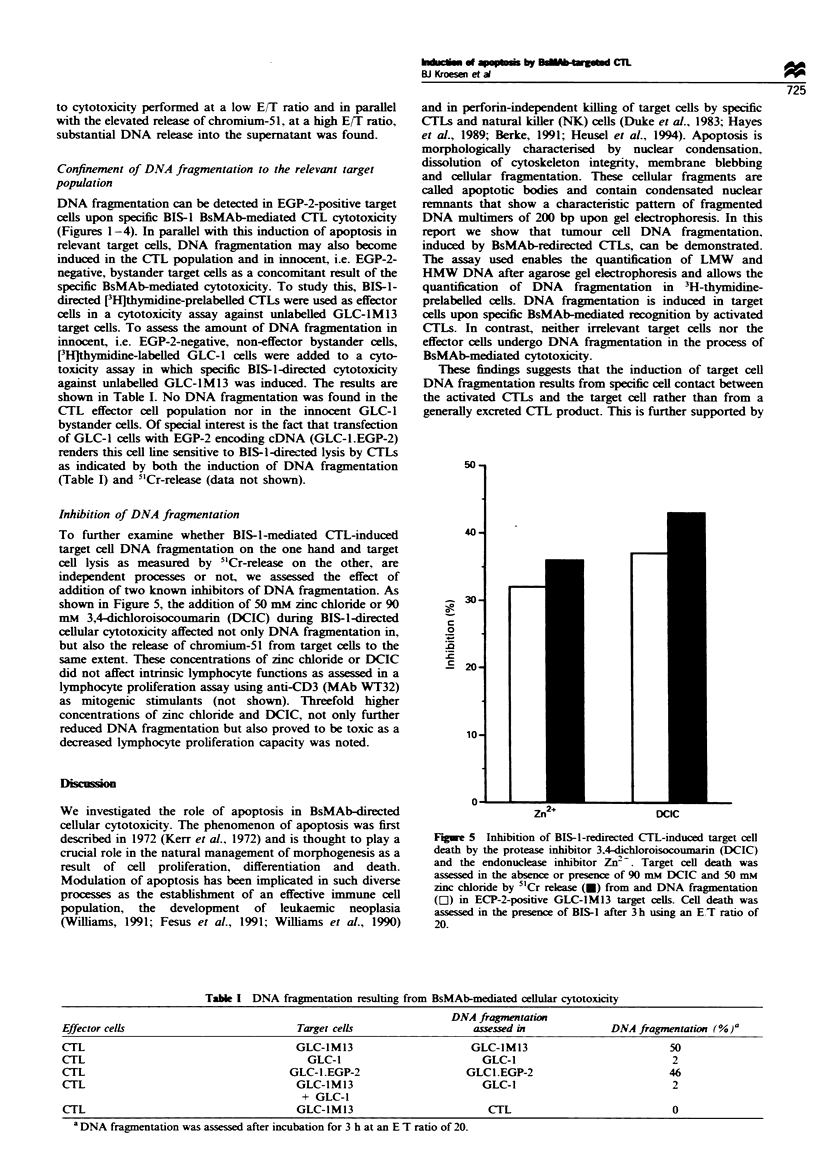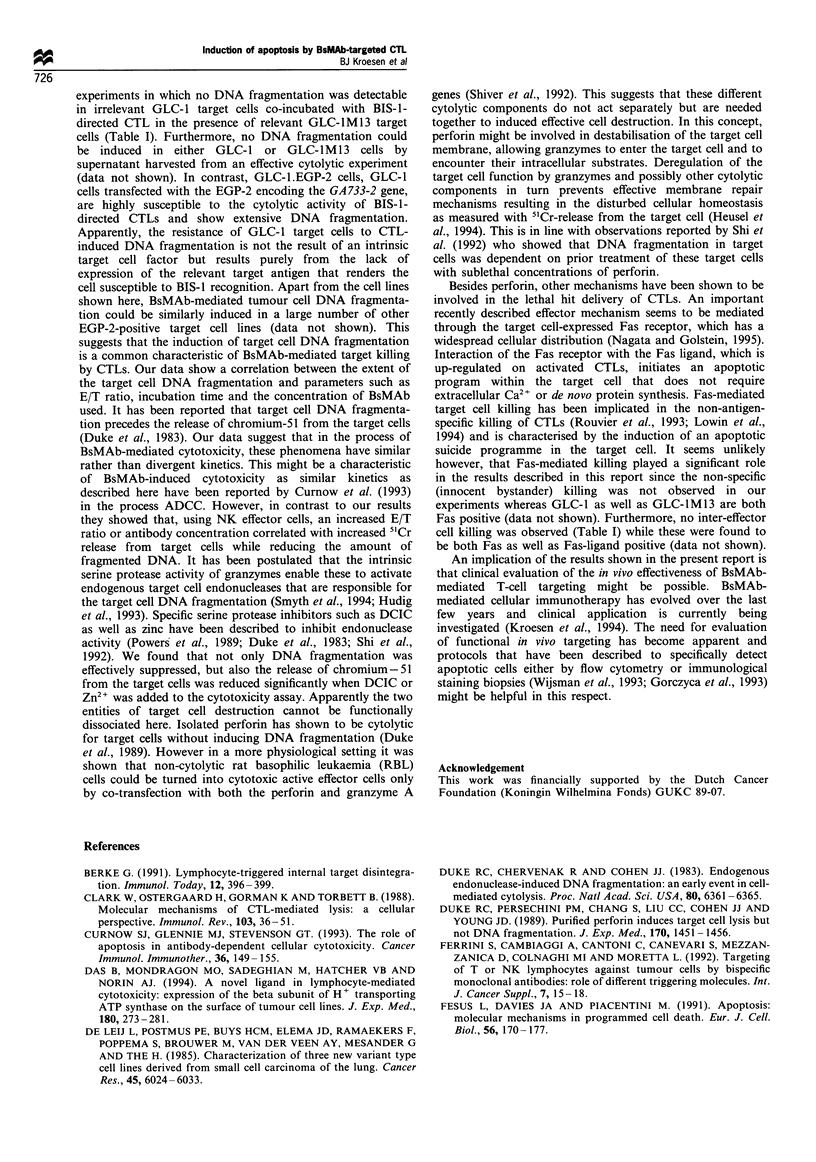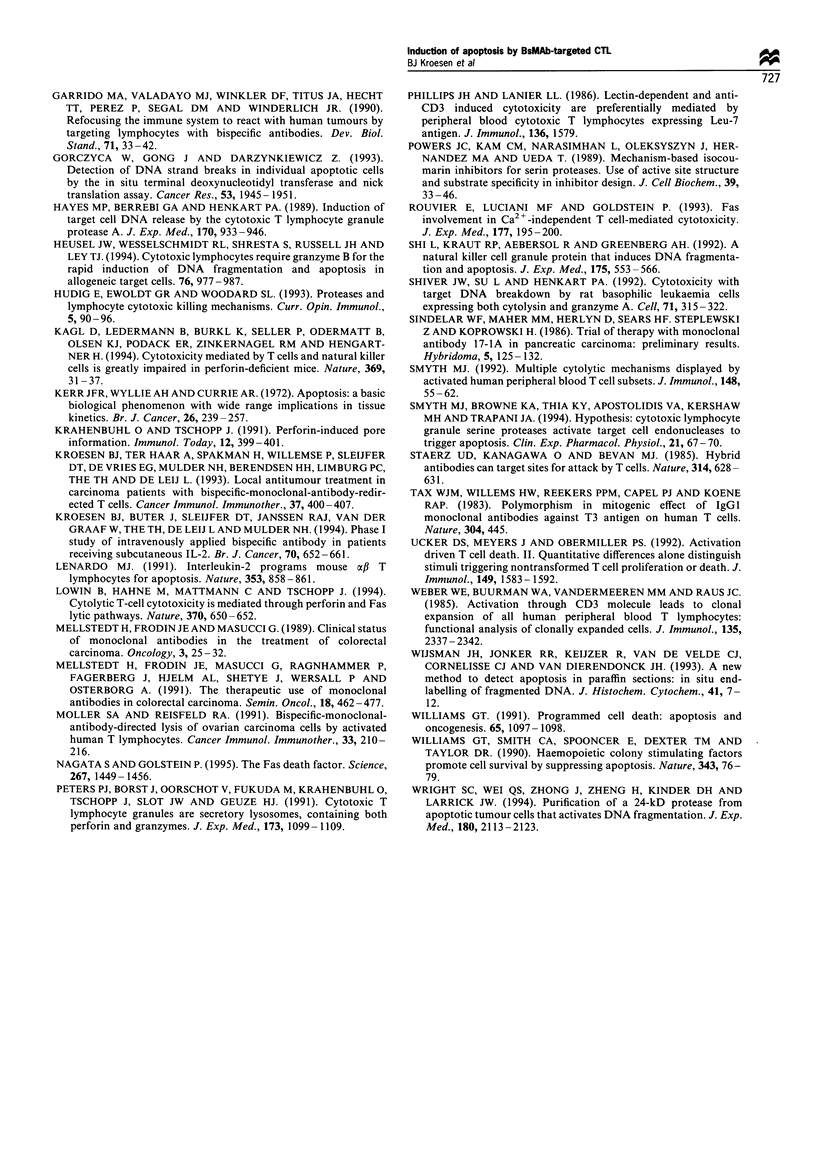# The role of apoptosis in bispecific antibody-mediated T-cell cytotoxicity.

**DOI:** 10.1038/bjc.1996.127

**Published:** 1996-03

**Authors:** B. J. Kroesen, G. J. Wellenberg, A. Bakker, W. Helfrich, T. H. The, L. de Leij

**Affiliations:** Department of Clinical Immunology, University Hospital Groningen, The Netherlands.

## Abstract

**Images:**


British Journal of Cancer (1996) 73, 721-727
© 1996 Stockton Press All rights reserved 0007-0920/96 $12.00

### The role of apoptosis in bispecific antibody-mediated T-cell cytotoxicity

BJ Kroesen, GJ Wellenberg, A Bakker, W Helfrich, TH The and L de Leij

*Department of Clinical Immunology, University Hospital Groningen, Oostersingel 59, 9713 EZ Groningen, The Netherlands.*

**Summary** In this report we describe the role of apoptosis in the process of tumour cell killing by bispecific monoclonal antibody (BsMAb)-redirected cytolytic T cells. The BsMAb used, BIS-1, has dual specificity for the CD3 complex on T cells and the pancarcinoma-associated 38 kDa transmembrane antigen EGP-2. BIS-1 allows activated T cells to specifically recognise and kill EGP-2-positive but not EGP-2-negative target cells. An assay was developed to quantify apoptosis in cells by separation of $^{3}H$-thymidine-labelled low-molecular, i.e. fragmented, from high-molecular, i.e. non-fragmented DNA. The presence of low molecular weight DNA was measured both within the target cells and in the cell-free supernatant. After exposure to BIS-1-redirected, -activated T cells, apoptosis was observed in EGP-2-positive target cells but not in EGP-2-negative target cells. Also no DNA fragmentation proved to be induced in the activated effector cells during assay. The degree of EGP-2-positive target DNA fragmentation depended on the concentration of BsMAb, the E/T ratio and the incubation time. Using a low E/T ratio (1/1), DNA fragmentation in and $^{51}Cr$ release from target cells showed similar characteristics and kinetics. At higher E/T ratio (20/1), the $^{51}Cr$ release from the target cells increased to a greater extent than the percentage fragmented target cell DNA. Inhibitors of DNA fragmentation added to the cytotoxicity assay inhibited not only DNA fragmentation, but also the release of chromium-51 from the target cells, suggesting that apoptosis and cell lysis are closely related in BsMAb-mediated cell killing.

**Keywords:** apoptosis; bispecific antibody; T-cell targeting; carcinoma

Activated cytotoxic T lymphocytes (CTLs) kill opposing target cells upon MHC class I restricted recognition. Direct bispecific monoclonal antibody (BsMAb)-mediated linking of some cell-surface molecules present on the CTLs with cell-surface molecules present on a target cell can direct the lethal hit of the CTLs towards a prechosen target cell population (Staerz *et al.*, 1985; Garrido *et al.*, 1990; Ferrini *et al.*, 1992). This BsMAb-redirected lysis is conducted irrespective of MHC class I expression by the target cells and T-cell receptor (TcR) specificity of the CTLs (Garrido *et al.*, 1990). Presumably the most potent T-cell surface structure that can be used to redirect the CTL activity is the CD3/TcR complex as present on all T lymphocytes (Garrido *et al.*, 1990; Ferrini *et al.*, 1992).

CTLs may kill target cells by various mechanisms. The exact pathways or possible interplay between these mechanisms is as yet not fully understood (Berke, 1991; Krahenbuhl and Tschopp, 1991; Smyth *et al.*, 1994; Smyth, 1992; Lowin *et al.*, 1994). CTLs contain granules in which a number of lytic molecules have been characterised. These include perforin or lymphotoxin, serine esterase or granzymes and proteoglycans. Proteoglycans are not lytic by themselves but are able to bind other lytic components and are thus thought to play a role in the deposition of the lytic constituents at the interface between the effector and target cell. Isolated perforin has been shown to be cytotoxic as it inserts itself in the cell membrane followed by polymerisation to form tubular structures that allow uncontrolled passage of small electrolytes and possibly larger macromolecules resulting in cell death by disturbed cellular homeostasis (Duke *et al.*, 1989). Both the insertion in the cell membrane and the polymerisation to transmembrane channels is dependent on the presence of $Ca^{2+}$. The CTL-associated serine esterases or granzymes belong to a family of related serine proteases with a variety of substrate specificities (Peters *et al.*, 1991; Das *et al.*, 1994; Hayes *et al.*, 1989; Wright *et al.*, 1994; Krahenbuhl and Tschopp, 1991; Duke *et al.*, 1989). Granzymes co-localise with perforin in the lytic granules and have been shown to induce target cell DNA fragmentation, possibly by activation of intracellular endonucleases (Hayes *et al.*, 1989; Smyth *et al.*, 1994; Hudig *et al.*, 1993).

Although perforin is highly cytolytic by itself in the presence of free extracellular $Ca^{2+}$, target cell lysis is observed also under $Ca^{2+}$-free conditions (Clark *et al.*, 1988; Rouvier *et al.*, 1993). Furthermore, target cell lysis, although only to a minor extent, can be established using perforin-deficient CTLs (Kagl *et al.*, 1994; Lowin *et al.*, 1994). These observations indicate the possibility that multiple lytic mechanisms can be displayed by CTLs to kill target cells. Indeed, it was recognised by Duke *et al.* (1983) that, in addition to target cell membrane damage, the induction of low molecular DNA, characteristic of apoptosis, was detectable during the process of cellular cytotoxicity. One particular problem in assessing target cell apoptosis is the fact that induction of DNA fragmentation may occur not only in the target cell, but also in the effector cell population (Lenardo, 1991; Ucker *et al.*, 1992). To be able to identify the nature of the assessed fragmented DNA, prelabelling of the DNA of the cell population of interest can be done. In the present report we have investigated the role of DNA fragmentation in BsMAb-mediated anti-tumour activity by detection and quantification of low molecular DNA using a combination of $^{3}H$-thymidine-prelabelling and agarose gel electrophoresis. BsMAb-mediated DNA fragmentation induced by BIS-1-redirected activated CTLs was assessed in EGP-2-positive and EGP-2-negative target cells and compared with cell death as a result of cell membrane damage as assessed in a classical $^{51}Cr$-release assay. The BsMAb BIS-1 recognises both the CD3 complex on T lymphocytes and the epithelial-related, pancarcinoma-associated 38 kDa transmembrane antigen EGP-2. EGP-2 has been described in a number of clinical trials as target antigen for site-directed immunotherapy of carcinomas using monoclonal- and BsMAb-based immunotherapeutical modalities (Kroesen *et al.*, 1994; Moller and Reisfeld, 1991; Sindelar *et al.*, 1986; Mellstedt *et al.*, 1989; 1991). Our results show that the different mechanisms of BIS-1-mediated target cell lysis are closely linked.

Correspondence: L de Leij, Department of Clinical Immunology, Internal Medicine, University Hospital Groningen, Oostersingel 59, 9713EZ Groningen, The Netherlands
Received 24 May 1995; revised 25 October 1995; accepted 14 November 1995

### Materials and methods

*Target cell lines*

GLC-1 (EGP-2 negative) and a clonal derivative thereof, GLC-1M13 (EGP-2 positive), are human small-cell lung cancer (SCLC)-derived cel lines (De Leij *et al.*, 1985). The cell lines were cultured according to routine procedures in culture medium, i.e. RPMI-1640 (Gibco/BRL, Paisley, UK) supplemented with 14% heat-inactivated fetal calf serum (Gibco/BRL), 2 mM glutamine (Gibco/BRL), 60 $\mu g\ ml^{-1}$ gentamicin (Schering, Kenilworth, USA), 0.05 mM $\beta$-mercaptoethanol (Merck, Darmstadt, Germany) and 1 mM sodium pyruvate (Gibco/BRL) at 37°C in a humidified atmosphere containing 5% carbon dioxide.

*CTL isolation and activation*

Peripheral blood mononuclear cells (PBMCs) were obtained from heparinised peripheral blood. Isolation was done by density centrifugation of diluted (1:1 in phosphate-buffered saline; PBS) blood on lymphoprep (Nycomed, Oslo, Norway) at 2400 r.p.m. for 20 min. The PBMC fraction was washed twice by resuspension in RPMI-1640 and centrifugation at 1800 (first time) and 1200 (second time) r.p.m. for 10 min. After isolation, PBMCs were collected in complete medium consisting of RPMI-1640 supplemented with 2% heat-inactivated human pooled serum, 2 mM gluatamine and 60 $\mu g\ ml^{-1}$ gentamicin. The CTL effector cells were prepared by *in vitro* T-cell activation, which was done by incubating the PBMCs for 3 days in complete medium supplemented with 5% (giving about 0.5 $\mu g\ ml^{-1}$ IgG $ml^{-1}$ end concentration) culture supernatant of the mitogenic anti-CD3 MAb WT-32 (Tax *et al.*, 1983), followed by washing and incubation for 2 additional days in complete medium supplemented with 100 IU $ml^{-1}$ interleukin 2 (IL-2) (EuroCetus, Amsterdam, The Netherlands).

*Bispecific antibody*

The BsMAb BIS-1 was made and purified as described (Kroesen *et al.*, 1993). In short, the BIS-1-producing quadroma was made by fusion of the hybridomas RIV-9 and MOC-31, producing anti-CD3 (IgG3) and anti-EGP-2 (IgG1) antibodies respectively. Purification of the hybrid antibodies (IgG3/IgG1) from parental-type antibodies (IgG3 and IgG1), also produced by the quadroma, was done by Protein A (Pharmacia, Uppsala, Sweden) column chromatography. Hollow fibre BIS-1 quadroma culture supernatant was loaded onto the column at pH 7.3 and the different IgG fractions were eluted successively by lowering the pH stepwise. The purified BIS-1 was tested for its bispecific characteristics both immunohistochemically and functionally in cytotoxicity assays.

*DNA fragmentation assay*

A DNA fragmentation assay was developed based on a modified procedure described by Duke *et al.* (1983) and Curnow *et al.* (1993). Modifications were the use of [$^3$H]-thymidine instead of [5-$^{125}$I]iodo-2′-deoxyuridine for labelling target cells and the introduction of an additional control experiment from which the degree of spontaneous target cell DNA fragmentation could be established. Before the assay, $5\times10^6$ target cells were labelled for 16 h at 37°C, 5% carbon dioxide in 1 ml of fresh culture medium containing 10 $\mu$Ci of [$^3$H]-thymidine (Amersham, Little Chalfont, UK). Unbound label was removed by washing the cells four times with culture medium. Aliquots of 50 $\mu l$ of culture medium containing $5\times10^4$ target cells were pipetted into each well of a 96-well round-bottom plate (Costar, Cambridge, MA, USA). Subsequently, 50 $\mu l$ of culture medium containing various amounts of BIS-1, IL-2 (400 IU $ml^{-1}$) and 100 $\mu l$ of CTL effector cells were added to each well to give the desired final BIS-1 concentration and effector to target ratio in a final volume of 200 $\mu l$ per well. The endonuclease inhibitors zinc chloride (Merck) and 3,4-dichloroisocoumarin (Sigma Chemical Co, St Louis, MO, USA) were added to the assay together with BIS-1 at the indicated concentrations. All determinations were done in quadruplicate. The microtitre plates were centrifuged at 500 r.p.m. for 2 min to initiate cell–cell contact and incubated at 37°C in 5% carbon dioxide for the indicated times. After the incubation, the plates were centrifuged at 1000 r.p.m. for 5 min and the contents of the four wells of each quadruplicate were pooled in an Eppendorf vial. Cells were pelleted by centrifugation at 13 000 r.p.m. for 5 min after which 100 $\mu l$ aliquots of the supernatants were mixed with 1 ml of Hisafe scintillation fluid (LKB Pharmacia, Uppsala, Sweden) and counted using a scintillation counter. The rest of the supernatant was discarded and the cell pellets were mixed and lysed in 60 $\mu l$ of lysis buffer containing 0.5% sodium-*N*-lauroylsarkosine (Sigma), 0.5 mg $ml^{-1}$ RNAase (Boehringer Mannheim, Germany), 1 mg $ml^{-1}$ proteinase K (Pharmacia) in 50 mM Tris-HCl, pH 8.0. After incubation for 2 h at 50°C, 30 $\mu l$ of the lysed pellet suspension was removed, mixed with 1 ml of Hisafe scintillation fluid and counted using a scintillation counter.

*Quantification of DNA fragmentation*

Visualisation and quantification of DNA fragmentation was done after separation by agarose gel electrophoresis. An aliquot of 7 $\mu l$ of gel electrophoresis loading buffer (0.04% bromophenol blue, 0.06% xylene cyanol FF and 20% Ficoll 400) was added to 30 $\mu l$ of lysed cell pellet suspension and mixed. This mixture was then loaded into dry wells of a 1.5% LMP agarose gel (Gibco/BRL) containing 0.5 $\mu g\ ml^{-1}$ ethidium bromide. After sample loading, electrophoresis was performed for 2–3 h at 100 V in TAE buffer (40 mM Tris-acetate, 1 mM EDTA). DNA was visualised by UV

**Figure 1** DNA fragmentation as a result of cytolytic activity of activated CTLs against GLC-1M13 (lanes 2 and 3) and GLC-1 (lanes 4 and 5) target cells in the presence (lanes 2 and 4) or absence (lanes 3 and 5) of the BsMAb BIS-1. BIS-1-targeted activated CTLs induce DNA fragmentation exclusively in EGP-2-positive GLC-1M13 target cells. CTLs incubated with BIS-1 do not undergo DNA fragmentation (lane 1). DNA fragmentation was assessed after 3 h at an E/T ratio of 20.

translumination. Quantification of DNA fragmentation was done by segmentation of the gel into individual lanes followed by dissecting the high and low molecular weight DNA containing gel sections (HMW DNA and LMW DNA respectively as exemplified in Figure 1). Approximately 50% (v/v) sodium hypochlorite solution was added to the gel sections in 20 ml glass vials (Packard, Groningen, The Netherlands) and the agarose was allowed to dissolve at 70°C. The samples containing HMW DNA were subsequently mixed with 5 ml of Hisafe, the samples containing LMW DNA were mixed with 15 ml of Hisafe scintillation fluid and counted in a scintillation counter. Samples were counted for 1 min and disintegrations per second (d.p.s.) were used in the following formulas to quantify the percentage DNA fragmentation.

Quantification of the percentage DNA fragmentation in the cell pellet:

$$\frac{(\text{LMW DNA})\text{exp.} - (\text{LMW DNA})\text{spont.}}{(\text{HMW DNA} + \text{LMW DNA})\text{exp.} - (\text{LMW DNA})\text{spont.}} \times 100\%$$

Quantification of the total percentage DNA fragmentation (cell pellet plus released DNA):

$$\frac{(\text{sup.} + \text{LMW DNA pell.})\text{exp.} - (\text{sup.} + \text{LMW DNA pell.})\text{spont.}}{(\text{sup.} + \text{HMW DNA pell.} + \text{LMW DNA pell.})\text{exp.} - (\text{sup.} + \text{LMW DNA pell.})\text{spont.}} \times 100\%$$

In the above formulas: sup. = the amount of d.p.s. assessed in the supernatant and pell. = the amount of d.p.s. assessed in the cell pellet. The spontaneous DNA fragmentation and DNA release was determined from a sample to which medium was added instead of effector cells.

### *$^{51}$Cr-release assay*

$^{51}$Cr-release assays were performed according to standard procedures to asses BIS-1-redirected T-cell cytotoxicity. All determinations were done in triplicate in the presence of 60 IU ml$^{-1}$ IL-2. Before the assay, $5 \times 10^6$ target cells (GLC-1M13 or GLC-1) were suspended in 100 $\mu$l culture medium containing 3.7 MBq $Na_2{}^{51}CrO_4$ (Amersham) and incubated for 1 h at 37°C, in a humidified, 5% carbon dioxide-containing atmosphere. Unbound $Na_2{}^{51}CrO_4$ was removed by washing the cells three times with medium. Aliquots of 100 $\mu$l of medium containing $2.5 \times 10^3$ $^{51}$Cr-labelled target cells were pipetted into each well of a 96-well round-bottom microtitre plate. Subsequently, 50 $\mu$l of medium containing various amounts of BIS-1 and 50 $\mu$l CTL effector cells was added to each well to give the desired final BIS-1 concentration and effector to target ratio in a final volume of 200 $\mu$l per well. The microtitre plates were centrifuged at 500 r.p.m. for 2 min and incubated at 37°C, 5% carbon dioxide for the indicated times. After the incubation, the plates were centrifuged at 1000 r.p.m. for 5 min and 100 $\mu$l samples taken from the supernatant were counted in an LKBG gamma counter (LKB Pharmacia) for 5 min. Cell lysis was calculated from the percentage $^{51}$Cr released, according to the formula:

$$\frac{\text{Experimental release} - \text{spontaneous release}}{\text{Maximal release} - \text{spontaneous release}} \times 100\%$$

Maximal release was determined from a sample to which 100 $\mu$l of 2% Triton X-100 solution was added instead of BIS-1 and effector cells. Spontaneous release was determined from a sample to which 50 $\mu$l of medium was added instead of effector cells.

### Results

### *Target cell DNA fragmentation after BsMAb-redirected T-cell cytotoxicity*

EGP-2-positive target cells showed apoptosis as a result of BsMAb-redirected CTL-mediated cytotoxicity. In Figure 1 DNA laddering with DNA fragments of 200 basepair multimers, characteristic of apoptosis, is visualised after specific, BIS-1-directed cytotoxicity against the target cell lines GLC-1M13 (EGP-2-positive) whereas no such laddering could be established in GLC-1 (EGP-2-negative) cells, in the CTL population alone, or in the absence of BIS-1.

**Figure 2** Kinetics of target cell death assessed by $^{51}$Cr release (●), DNA fragmentation inside the target cells (△) and total target cell DNA fragmentation (▽). Cytolytic activity was assessed after 3 h at various E/T ratios in the presence of the BsMAb BIS-1 against EGP-2-positive GLC-1M13 target cells (**a**) and EGP-2-negative GLC-1 target cells (**b**). Mean values ± s.e. are shown.

Quantification of $^{3}$H-thymidine-prelabelled DNA fragmentation was done by segmentation of the gel into individual lanes and separation of HMW DNA from LMW DNA-containing parts of the lane followed by scintillation counting. By also counting the amount of DNA (fragments) released into the supernatant during the assay, DNA degradation could be differentiated into DNA fragmentation inside in as yet intact target cells and the total percentage of DNA fragmentation. The CTLs, used as the effector cell population in the experiments shown below, were generated from PBMCs by an activation protocol described to yield a cytolytic effector cell population composed of predominantly CD8-positive T lymphocytes (Weber *et al.*, 1985; Phillips and Lanier, 1986). Figures 2, 3 and 4 show the percentage DNA fragmentation in GLC-1M13 and GLC-1 target cells as a result of BIS-1-redirected CTL activity. DNA fragmentation was always compared with the results of a simultaneously performed $^{51}$Cr-release assay. The percentage target cell DNA fragmentation proved to be dependent on the E/T ratio used (Figure 2), the amount of BIS-1 added to the assay (Figure 3) and the incubation time (Figure 4). Specific DNA fragmentation was found in the EGP-2-positive GLC-1M13 target cells (Figure 2a) but not the EGP-2-negative GLC-1 target cell (Figure 2b), which is in agreement with the qualitative data shown in Figure 1. Fragmented DNA was found not only within the target cells but also in the supernatant, resulting in an increased total DNA fragmentation compared with the DNA fragmentation assessable within the target cells. Higher E/T ratios resulted in an increased fragmentation of target cell DNA, although chromium-51 release from the target cells appeared to increase to an even larger extent. Elevated target cell killing, as reflected by DNA fragmentation, was observed also as a result of increasing the concentration of BIS-1 in the cytotoxicity assay (Figure 3). It has been reported that DNA fragmentation precedes the release of chromium-51 from the target cells (Duke *et al.*, 1983). Using BIS-1-redirected CTLs, the time kinetics of DNA fragmentation proved to be essentially the same as those found with the $^{51}$Cr-release assay (Figure 4). Using a low E/T ratio (E/T = 1), the percentage DNA fragmentation equalled the percentage $^{51}$Cr-release at each of the assessed time points up to 180 min (Figure 4b). However, using a high E/T ratio (E/T = 20), the percentage $^{51}$Cr-release increased more rapidly in time than the percentage DNA fragmentation. Furthermore, in contrast

**Figure 3** Cytolytic activity of activated, BIS-1-redirected, CTLs against GLC-1M13 cells. $^{51}$Cr release (●), DNA fragmentation inside the target cells (△) and total target cell DNA fragmentation (▽) were assessed at various concentrations of BIS-1 after 3 h at an E/T ratio of 20. Mean values ± s.e. are shown.

**Figure 4** Kinetics of target cell death assessed by $^{51}$Cr release (●), DNA fragmentation inside the target cells (△) and total target cell DNA fragmentation (▽). Cytolytic activity was assessed after various incubation times at a fixed E/T ratio of 20 in the presence of the BsMAb BIS-1 against EGP-2-positive GLC-1M13 target cells (**a**) and EGP-2-negative GLC-1 target cells (**b**). Mean values ± s.e. values are shown.

to cytotoxicity performed at a low E/T ratio and in parallel with the elevated release of chromium-51, at a high E/T ratio, substantial DNA release into the supernatant was found.

*Confinement of DNA fragmentation to the relevant target population*

DNA fragmentation can be detected in EGP-2-positive target cells upon specific BIS-1 BsMAb-mediated CTL cytotoxicity (Figures 1–4). In parallel with this induction of apoptosis in relevant target cells, DNA fragmentation may also become induced in the CTL population and in innocent, i.e. EGP-2-negative, bystander target cells as a concomitant result of the specific BsMAb-mediated cytotoxicity. To study this, BIS-1-directed [$^3$H]thymidine-prelabelled CTLs were used as effector cells in a cytotoxicity assay against unlabelled GLC-1M13 target cells. To assess the amount of DNA fragmentation in innocent, i.e. EGP-2-negative, non-effector bystander cells, [$^3$H]thymidine-labelled GLC-1 cells were added to a cytotoxicity assay in which specific BIS-1-directed cytotoxicity against unlabelled GLC-1M13 was induced. The results are shown in Table I. No DNA fragmentation was found in the CTL effector cell population nor in the innocent GLC-1 bystander cells. Of special interest is the fact that transfection of GLC-1 cells with EGP-2 encoding cDNA (GLC-1.EGP-2) renders this cell line sensitive to BIS-1-directed lysis by CTLs as indicated by both the induction of DNA fragmentation (Table I) and $^{51}$Cr-release (data not shown).

*Inhibition of DNA fragmentation*

To further examine whether BIS-1-mediated CTL-induced target cell DNA fragmentation on the one hand and target cell lysis as measured by $^{51}$Cr-release on the other, are independent processes or not, we assessed the effect of addition of two known inhibitors of DNA fragmentation. As shown in Figure 5, the addition of 50 mM zinc chloride or 90 mM 3,4-dichloroisocoumarin (DCIC) during BIS-1-directed cellular cytotoxicity affected not only DNA fragmentation in, but also the release of chromium-51 from target cells to the same extent. These concentrations of zinc chloride or DCIC did not affect intrinsic lymphocyte functions as assessed in a lymphocyte proliferation assay using anti-CD3 (MAb WT32) as mitogenic stimulants (not shown). Threefold higher concentrations of zinc chloride and DCIC, not only further reduced DNA fragmentation but also proved to be toxic as a decreased lymphocyte proliferation capacity was noted.

### Discussion

We investigated the role of apoptosis in BsMAb-directed cellular cytotoxicity. The phenomenon of apoptosis was first described in 1972 (Kerr *et al.*, 1972) and is thought to play a crucial role in the natural management of morphogenesis as a result of cell proliferation, differentiation and death. Modulation of apoptosis has been implicated in such diverse processes as the establishment of an effective immune cell population, the development of leukaemic neoplasia (Williams, 1991; Fesus *et al.*, 1991; Williams *et al.*, 1990) and in perforin-independent killing of target cells by specific CTLs and natural killer (NK) cells (Duke *et al.*, 1983; Hayes *et al.*, 1989; Berke, 1991; Heusel *et al.*, 1994). Apoptosis is morphologically characterised by nuclear condensation, dissolution of cytoskeleton integrity, membrane blebbing and cellular fragmentation. These cellular fragments are called apoptotic bodies and contain condensated nuclear remnants that show a characteristic pattern of fragmented DNA multimers of 200 bp upon gel electrophoresis. In this report we show that tumour cell DNA fragmentation, induced by BsMAb-redirected CTLs, can be demonstrated. The assay used enables the quantification of LMW and HMW DNA after agarose gel electrophoresis and allows the quantification of DNA fragmentation in $^3$H-thymidine-prelabelled cells. DNA fragmentation is induced in target cells upon specific BsMAb-mediated recognition by activated CTLs. In contrast, neither irrelevant target cells nor the effector cells undergo DNA fragmentation in the process of BsMAb-mediated cytotoxicity.

These findings suggests that the induction of target cell DNA fragmentation results from specific cell contact between the activated CTLs and the target cell rather than from a generally excreted CTL product. This is further supported by

**Figure 5** Inhibition of BIS-1-redirected CTL-induced target cell death by the protease inhibitor 3,4-dichloroisocoumarin (DCIC) and the endonuclease inhibitor $Zn^{2+}$. Target cell death was assessed in the absence or presence of 90 mM DCIC and 50 mM zinc chloride by $^{51}$Cr release (■) from and DNA fragmentation (□) in ECP-2-positive GLC-1M13 target cells. Cell death was assessed in the presence of BIS-1 after 3 h using an E/T ratio of 20.

**Table I** DNA fragmentation resulting from BsMAb-mediated cellular cytotoxicity

| *Effector cells* | *Target cells* | *DNA fragmentation assessed in* | *DNA fragmentation (%)[a]* |
|---|---|---|---|
| CTL | GLC-1M13 | GLC-1M13 | 50 |
| CTL | GLC-1 | GLC-1 | 2 |
| CTL | GLC-1.EGP-2 | GLC1.EGP-2 | 46 |
| CTL | GLC-1M13 + GLC-1 | GLC-1 | 2 |
| CTL | GLC-1M13 | CTL | 0 |

[a] DNA fragmentation was assessed after incubation for 3 h at an E/T ratio of 20.

experiments in which no DNA fragmentation was detectable in irrelevant GLC-1 target cells co-incubated with BIS-1-directed CTL in the presence of relevant GLC-1M13 target cells (Table I). Furthermore, no DNA fragmentation could be induced in either GLC-1 or GLC-1M13 cells by supernatant harvested from an effective cytolytic experiment (data not shown). In contrast, GLC-1.EGP-2 cells, GLC-1 cells transfected with the EGP-2 encoding the *GA733-2* gene, are highly susceptible to the cytolytic activity of BIS-1-directed CTLs and show extensive DNA fragmentation. Apparently, the resistance of GLC-1 target cells to CTL-induced DNA fragmentation is not the result of an intrinsic target cell factor but results purely from the lack of expression of the relevant target antigen that renders the cell susceptible to BIS-1 recognition. Apart from the cell lines shown here, BsMAb-mediated tumour cell DNA fragmentation could be similarly induced in a large number of other EGP-2-positive target cell lines (data not shown). This suggests that the induction of target cell DNA fragmentation is a common characteristic of BsMAb-mediated target killing by CTLs. Our data show a correlation between the extent of the target cell DNA fragmentation and parameters such as E/T ratio, incubation time and the concentration of BsMAb used. It has been reported that target cell DNA fragmentation precedes the release of chromium-51 from the target cells (Duke *et al.*, 1983). Our data suggest that in the process of BsMAb-mediated cytotoxicity, these phenomena have similar rather than divergent kinetics. This might be a characteristic of BsMAb-induced cytotoxicity as similar kinetics as described here have been reported by Curnow *et al.* (1993) in the process ADCC. However, in contrast to our results they showed that, using NK effector cells, an increased E/T ratio or antibody concentration correlated with increased $^{51}$Cr release from target cells while reducing the amount of fragmented DNA. It has been postulated that the intrinsic serine protease activity of granzymes enable these to activate endogenous target cell endonucleases that are responsible for the target cell DNA fragmentation (Smyth *et al.*, 1994; Hudig *et al.*, 1993). Specific serine protease inhibitors such as DCIC as well as zinc have been described to inhibit endonuclease activity (Powers *et al.*, 1989; Duke *et al.*, 1983; Shi *et al.*, 1992). We found that not only DNA fragmentation was effectively suppressed, but also the release of chromium−51 from the target cells was reduced significantly when DCIC or $Zn^{2+}$ was added to the cytotoxicity assay. Apparently the two entities of target cell destruction cannot be functionally dissociated here. Isolated perforin has shown to be cytolytic for target cells without inducing DNA fragmentation (Duke *et al.*, 1989). However in a more physiological setting it was shown that non-cytolytic rat basophilic leukaemia (RBL) cells could be turned into cytotoxic active effector cells only by co-transfection with both the perforin and granzyme A genes (Shiver *et al.*, 1992). This suggests that these different cytolytic components do not act separately but are needed together to induced effective cell destruction. In this concept, perforin might be involved in destabilisation of the target cell membrane, allowing granzymes to enter the target cell and to encounter their intracellular substrates. Deregulation of the target cell function by granzymes and possibly other cytolytic components in turn prevents effective membrane repair mechanisms resulting in the disturbed cellular homeostasis as measured with $^{51}$Cr-release from the target cell (Heusel *et al.*, 1994). This is in line with observations reported by Shi *et al.* (1992) who showed that DNA fragmentation in target cells was dependent on prior treatment of these target cells with sublethal concentrations of perforin.

Besides perforin, other mechanisms have been shown to be involved in the lethal hit delivery of CTLs. An important recently described effector mechanism seems to be mediated through the target cell-expressed Fas receptor, which has a widespread cellular distribution (Nagata and Golstein, 1995). Interaction of the Fas receptor with the Fas ligand, which is up-regulated on activated CTLs, initiates an apoptotic program within the target cell that does not require extracellular $Ca^{2+}$ or *de novo* protein synthesis. Fas-mediated target cell killing has been implicated in the non-antigen-specific killing of CTLs (Rouvier *et al.*, 1993; Lowin *et al.*, 1994) and is characterised by the induction of an apoptotic suicide programme in the target cell. It seems unlikely however, that Fas-mediated killing played a significant role in the results described in this report since the non-specific (innocent bystander) killing was not observed in our experiments whereas GLC-1 as well as GLC-1M13 are both Fas positive (data not shown). Furthermore, no inter-effector cell killing was observed (Table I) while these were found to be both Fas as well as Fas-ligand positive (data not shown).

An implication of the results shown in the present report is that clinical evaluation of the *in vivo* effectiveness of BsMAb-mediated T-cell targeting might be possible. BsMAb-mediated cellular immunotherapy has evolved over the last few years and clinical application is currently being investigated (Kroesen *et al.*, 1994). The need for evaluation of functional *in vivo* targeting has become apparent and protocols that have been described to specifically detect apoptotic cells either by flow cytometry or immunological staining biopsies (Wijsman *et al.*, 1993; Gorczyca *et al.*, 1993) might be helpful in this respect.

**Acknowledgement**
This work was financially supported by the Dutch Cancer Foundation (Koningin Wilhelmina Fonds) GUKC 89-07.

### References

BERKE G. (1991). Lymphocyte-triggered internal target disintegration. *Immunol. Today*, **12**, 396–399.

CLARK W, OSTERGAARD H, GORMAN K AND TORBETT B. (1988). Molecular mechanisms of CTL-mediated lysis: a cellular perspective. *Immunol. Rev.*, **103**, 36–51.

CURNOW SJ, GLENNIE MJ, STEVENSON GT. (1993). The role of apoptosis in antibody-dependent cellular cytotoxicity. *Cancer Immunol. Immunother.*, **36**, 149–155.

DAS B, MONDRAGON MO, SADEGHIAN M, HATCHER VB AND NORIN AJ. (1994). A novel ligand in lymphocyte-mediated cytotoxicity: expression of the beta subunit of $H^+$ transporting ATP synthase on the surface of tumour cell lines. *J. Exp. Med.*, **180**, 273–281.

DE LEIJ L, POSTMUS PE, BUYS HCM, ELEMA JD, RAMAEKERS F, POPPEMA S, BROUWER M, VAN DER VEEN AY, MESANDER G AND THE H. (1985). Characterization of three new variant type cell lines derived from small cell carcinoma of the lung. *Cancer Res.*, **45**, 6024–6033.

DUKE RC, CHERVENAK R AND COHEN JJ. (1983). Endogenous endonuclease-induced DNA fragmentation: an early event in cell-mediated cytolysis. *Proc. Natl Acad. Sci. USA*, **80**, 6361–6365.

DUKE RC, PERSECHINI PM, CHANG S, LIU CC, COHEN JJ AND YOUNG JD. (1989). Purified perforin induces target cell lysis but not DNA fragmentation. *J. Exp. Med.*, **170**, 1451–1456.

FERRINI S, CAMBIAGGI A, CANTONI C, CANEVARI S, MEZZANZANICA D, COLNAGHI MI AND MORETTA L. (1992). Targeting of T or NK lymphocytes against tumour cells by bispecific monoclonal antibodies: role of different triggering molecules. *Int. J. Cancer Suppl.*, **7**, 15–18.

FESUS L, DAVIES JA AND PIACENTINI M. (1991). Apoptosis: molecular mechanisms in programmed cell death. *Eur. J. Cell. Biol.*, **56**, 170–177.

GARRIDO MA, VALADAYO MJ, WINKLER DF, TITUS JA, HECHT TT, PEREZ P, SEGAL DM AND WINDERLICH JR. (1990). Refocusing the immune system to react with human tumours by targeting lymphocytes with bispecific antibodies. *Dev. Biol. Stand.*, **71,** 33–42.

GORCZYCA W, GONG J AND DARZYNKIEWICZ Z. (1993). Detection of DNA strand breaks in individual apoptotic cells by the in situ terminal deoxynucleotidyl transferase and nick translation assay. *Cancer Res.*, **53,** 1945–1951.

HAYES MP, BERREBI GA AND HENKART PA. (1989). Induction of target cell DNA release by the cytotoxic T lymphocyte granule protease A. *J. Exp. Med.*, **170,** 933–946.

HEUSEL JW, WESSELSCHMIDT RL, SHRESTA S, RUSSELL JH AND LEY TJ. (1994). Cytotoxic lymphocytes require granzyme B for the rapid induction of DNA fragmentation and apoptosis in allogeneic target cells. **76,** 977–987.

HUDIG E, EWOLDT GR AND WOODARD SL. (1993). Proteases and lymphocyte cytotoxic killing mechanisms. *Curr. Opin. Immunol.*, **5,** 90–96.

KAGL D, LEDERMANN B, BURKL K, SELLER P, ODERMATT B, OLSEN KJ, PODACK ER, ZINKERNAGEL RM AND HENGARTNER H. (1994). Cytotoxicity mediated by T cells and natural killer cells is greatly impaired in perforin-deficient mice. *Nature*, **369,** 31–37.

KERR JFR, WYLLIE AH AND CURRIE AR. (1972). Apoptosis: a basic biological phenomenon with wide range implications in tissue kinetics. *Br. J. Cancer*, **26,** 239–257.

KRAHENBUHL O AND TSCHOPP J. (1991). Perforin-induced pore information. *Immunol. Today*, **12,** 399–401.

KROESEN BJ, TER HAAR A, SPAKMAN H, WILLEMSE P, SLEIJFER DT, DE VRIES EG, MULDER NH, BERENDSEN HH, LIMBURG PC, THE TH AND DE LEIJ L. (1993). Local antitumour treatment in carcinoma patients with bispecific-monoclonal-antibody-redirected T cells. *Cancer Immunol. Immunother.*, **37,** 400–407.

KROESEN BJ, BUTER J, SLEIJFER DT, JANSSEN RAJ, VAN DER GRAAF W, THE TH, DE LEIJ L AND MULDER NH. (1994). Phase I study of intravenously applied bispecific antibody in patients receiving subcutaneous IL-2. *Br. J. Cancer*, **70,** 652–661.

LENARDO MJ. (1991). Interleukin-2 programs mouse $\alpha\beta$ T lymphocytes for apoptosis. *Nature*, **353,** 858–861.

LOWIN B, HAHNE M, MATTMANN C AND TSCHOPP J. (1994). Cytolytic T-cell cytotoxicity is mediated through perforin and Fas lytic pathways. *Nature*, **370,** 650–652.

MELLSTEDT H, FRODIN JE AND MASUCCI G. (1989). Clinical status of monoclonal antibodies in the treatment of colorectal carcinoma. *Oncology*, **3,** 25–32.

MELLSTEDT H, FRODIN JE, MASUCCI G, RAGNHAMMER P, FAGERBERG J, HJELM AL, SHETYE J, WERSALL P AND OSTERBORG A. (1991). The therapeutic use of monoclonal antibodies in colorectal carcinoma. *Semin. Oncol.*, **18,** 462–477.

MOLLER SA AND REISFELD RA. (1991). Bispecific-monoclonal-antibody-directed lysis of ovarian carcinoma cells by activated human T lymphocytes. *Cancer Immunol. Immunother.*, **33,** 210–216.

NAGATA S AND GOLSTEIN P. (1995). The Fas death factor. *Science*, **267,** 1449–1456.

PETERS PJ, BORST J, OORSCHOT V, FUKUDA M, KRAHENBUHL O, TSCHOPP J, SLOT JW AND GEUZE HJ. (1991). Cytotoxic T lymphocyte granules are secretory lysosomes, containing both perforin and granzymes. *J. Exp. Med.*, **173,** 1099–1109.

PHILLIPS JH AND LANIER LL. (1986). Lectin-dependent and anti-CD3 induced cytotoxicity are preferentially mediated by peripheral blood cytotoxic T lymphocytes expressing Leu-7 antigen. *J. Immunol.*, **136,** 1579.

POWERS JC, KAM CM, NARASIMHAN L, OLEKSYSZYN J, HERNANDEZ MA AND UEDA T. (1989). Mechanism-based isocoumarin inhibitors for serin proteases. Use of active site structure and substrate specificity in inhibitor design. *J. Cell Biochem.*, **39,** 33–46.

ROUVIER E, LUCIANI MF AND GOLDSTEIN P. (1993). Fas involvement in $Ca^{2+}$-independent T cell-mediated cytotoxicity. *J. Exp. Med.*, **177,** 195–200.

SHI L, KRAUT RP, AEBERSOL R AND GREENBERG AH. (1992). A natural killer cell granule protein that induces DNA fragmentation and apoptosis. *J. Exp. Med.*, **175,** 553–566.

SHIVER JW, SU L AND HENKART PA. (1992). Cytotoxicity with target DNA breakdown by rat basophilic leukaemia cells expressing both cytolysin and granzyme A. *Cell*, **71,** 315–322.

SINDELAR WF, MAHER MM, HERLYN D, SEARS HF. STEPLEWSKI Z AND KOPROWSKI H. (1986). Trial of therapy with monoclonal antibody 17-1A in pancreatic carcinoma: preliminary results. *Hybridoma*, **5,** 125–132.

SMYTH MJ. (1992). Multiple cytolytic mechanisms displayed by activated human peripheral blood T cell subsets. *J. Immunol.*, **148,** 55–62.

SMYTH MJ, BROWNE KA, THIA KY, APOSTOLIDIS VA, KERSHAW MH AND TRAPANI JA. (1994). Hypothesis: cytotoxic lymphocyte granule serine proteases activate target cell endonucleases to trigger apoptosis. *Clin. Exp. Pharmacol. Physiol.*, **21,** 67–70.

STAERZ UD, KANAGAWA O AND BEVAN MJ. (1985). Hybrid antibodies can target sites for attack by T cells. *Nature*, **314,** 628–631.

TAX WJM, WILLEMS HW, REEKERS PPM, CAPEL PJ AND KOENE RAP. (1983). Polymorphism in mitogenic effect of IgG1 monoclonal antibodies against T3 antigen on human T cells. *Nature*, **304,** 445.

UCKER DS, MEYERS J AND OBERMILLER PS. (1992). Activation driven T cell death. II. Quantitative differences alone distinguish stimuli triggering nontransformed T cell proliferation or death. *J. Immunol.*, **149,** 1583–1592.

WEBER WE, BUURMAN WA, VANDERMEEREN MM AND RAUS JC. (1985). Activation through CD3 molecule leads to clonal expansion of all human peripheral blood T lymphocytes: functional analysis of clonally expanded cells. *J. Immunol.*, **135,** 2337–2342.

WIJSMAN JH, JONKER RR, KEIJZER R, VAN DE VELDE CJ, CORNELISSE CJ AND VAN DIERENDONCK JH. (1993). A new method to detect apoptosis in paraffin sections: in situ end-labelling of fragmented DNA. *J. Histochem. Cytochem.*, **41,** 7–12.

WILLIAMS GT. (1991). Programmed cell death: apoptosis and oncogenesis. **65,** 1097–1098.

WILLIAMS GT, SMITH CA, SPOONCER E, DEXTER TM AND TAYLOR DR. (1990). Haemopoietic colony stimulating factors promote cell survival by suppressing apoptosis. *Nature*, **343,** 76–79.

WRIGHT SC, WEI QS, ZHONG J, ZHENG H, KINDER DH AND LARRICK JW. (1994). Purification of a 24-kD protease from apoptotic tumour cells that activates DNA fragmentation. *J. Exp. Med.*, **180,** 2113–2123.